# Comparison of assessment of diaphragm function using speckle tracking between patients with successful and failed weaning: a multicentre, observational, pilot study

**DOI:** 10.1186/s12890-022-02260-z

**Published:** 2022-12-01

**Authors:** Qiancheng Xu, Xiao Yang, Yan Qian, Chang Hu, Weihua Lu, Shuhan Cai, Bo Hu, Jianguo Li

**Affiliations:** 1 Department of Critical Care Medicine, Zhongnan Hospital of Wuhan University, Wuhan, 430071 Hubei China; 2 Clinical Research Center of Hubei Critical Care Medicine, Wuhan, 430071 Hubei China; 3 Department of Critical Care Medicine, The First Affiliated Hospital of Wannan Medical College (Yijishan Hospital of Wannan Medical College), Wuhu, 241000 Anhui China; 4 Anhui Provincial Clinical Research Center for Critical Respiratory Disease, Wuhu, 241000 Anhui China; 5 Department of Emergency Intensive Care Unit, EastChina Normal University, Wuhu Hospital, Wuhu, 241000 Anhui China

**Keywords:** Speckle tracking, Diaphragm, Ultrasound, Strain, Weaning, Mechanical ventilation

## Abstract

**Background:**

Diaphragmatic ultrasound has been increasingly used to evaluate diaphragm function. However, current diaphragmatic ultrasound parameters provide indirect estimates of diaphragmatic contractile function, and the predictive value is controversial. Two-dimensional (2D) speckle tracking is an effective technology for measuring tissue deformation and can be used to measure diaphragm longitudinal strain (DLS) to assess diaphragm function. The purpose of this study was to determine the feasibility and reproducibility of DLS quantification by 2D speckle tracking and to determine whether maximal DLS could be used to predict weaning outcomes.

**Methods:**

This study was performed in the intensive care unit of two teaching hospitals, and was divided into two studies. Study A was a prospective study to evaluate the feasibility, reliability, and repeatability of speckle tracking in assessing DLS in healthy subjects and mechanically ventilated patients. Study B was a multicentre retrospective study to assess the use of maximal DLS measured by speckle tracking in predicting weaning outcomes.

**Results:**

Twenty-five healthy subjects and twenty mechanically ventilated patients were enrolled in Study A. Diaphragmatic speckle tracking was easily accessible. The intra- and interoperator reliability were good to excellent under conditions of eupnoea, deep breathing, and mechanical ventilation. The intraclass correlation coefficient (ICC) ranged from 0.78 to 0.95. Ninety-six patients (fifty-nine patients were successfully weaned) were included in Study B. DLS exhibited a fair linear relationship with both the diaphragmatic thickening fraction (DTF) (R^2^ = 0.73, *p* < 0.0001) and diaphragmatic excursion (DE) (R^2^ = 0.61, *p* < 0.0001). For the prediction of successful weaning, the areas under the ROC curves of DLS, diaphragmatic thickening fraction DTF, RSBI, and DE were 0.794, 0.794, 0.723, and 0.728, respectively. The best cut-off value for predicting the weaning success of DLS was less than -21%, which had the highest sensitivity of 89.19% and specificity of 64.41%.

**Conclusions:**

Diaphragmatic strain quantification using speckle tracking is easy to obtain in healthy subjects and mechanically ventilated patients and has a high predictive value for mechanical weaning. However, this method offers no advantage over RSBI. Future research should assess its value as a predictor of weaning.

**Trial registration:**

This study was registered in the Chinese Clinical Trial Register (ChiCTR), ChiCTR2100049816. Registered 10 August 2021. http://www.chictr.org.cn/showproj.aspx?proj=131790

**Supplementary Information:**

The online version contains supplementary material available at 10.1186/s12890-022-02260-z.

## Background

Weaning from mechanical ventilation is one of the most common and difficult tasks for intensivists. Premature weaning may result in reintubation in up to 25% of patients [1, 2]. On the other hand, delayed extubation is associated with ventilator-induced diaphragmatic atrophy and ventilator-associated pneumonia and may lead to high morbidity and mortality, prolonged intensive care unit (ICU) length of stay, and increased health care costs [3–7]. Therefore, there is a need for a safe and effective method to aid in selecting the optimum timing to terminate mechanical ventilation. Respiratory mechanic parameters, including the rapid shallow breathing index (RSBI: the ratio of respiratory frequency to tidal volume) and maximum inspiratory pressure (MIP) [8], are typically used to predict weaning; however, the predictive value of these parameters is controversial. A recent meta-analysis showed that RSBI only had moderate sensitivity and poor specificity for predicting extubation success [9].

The diaphragm is the main inspiratory muscle and plays a major role in breathing [10]. Diaphragmatic dysfunction is a common cause of weaning failure in critically ill patients, as has been thoroughly documented [10–12]. Point-of-care diaphragmatic ultrasonography is the most popular method to assess the function of the diaphragm due to its portability, noninvasiveness, and ease of reproducibility [8, 11, 12]. Diaphragmatic excursion (DE), diaphragmatic thickness at end-inspiration (DTei), end-expiration (DTee), and diaphragmatic thickening fraction (DTF) are the most commonly used diaphragmatic ultrasound parameters. Although recent studies have shown that diaphragm ultrasound exhibits better weaning predictability than RSBI [13], current parameters remain insufficient. First, ultrasonic monitoring has high technical requirements, and the heterogeneity of technology will affect the judgement accuracy of physicians [14]. Second, parameters, such as DTF and DE, are indirect estimates of diaphragmatic contractile function as they do not assess muscle "longitudinal" shortening, i.e., motion along the "plane" of muscle fibre [15].

Two-dimensional (2D) speckle tracking is an effective method of measuring tissue deformation (‘strain’) and is most commonly used to assess cardiac function [16]. In recent years, 2D speckle tracking has been used to measure strain in variable tissues to assess their function, such as the diaphragm [15, 17, 18], skeletal muscle [19, 20], pleura [21, 22], tendon [23], and carpal tunnel [24]. Two-dimensional speckle tracking is mostly used in healthy volunteers to evaluate its feasibility, reliability, and repeatability in testing the function of tissues and organs at the moment; however, few studies have evaluated its clinical application value [15, 17, 18]. In particular, no study has been reported the use of 2D speckle tracking to assess the value of diaphragmatic function for predicting weaning.

Based on the above background statement, our study aimed to evaluate the feasibility, reliability, and repeatability of 2D speckle tracking in assessing diaphragmatic longitudinal strain (DLS) in healthy subjects and mechanically ventilated patients and to determine whether 2D speckle tracking quantification of DLS could be used to predict the outcome of weaning in mechanically ventilated patients compared to conventional diaphragmatic ultrasound parameters.

## Study design and methods

**Fig. 1:**
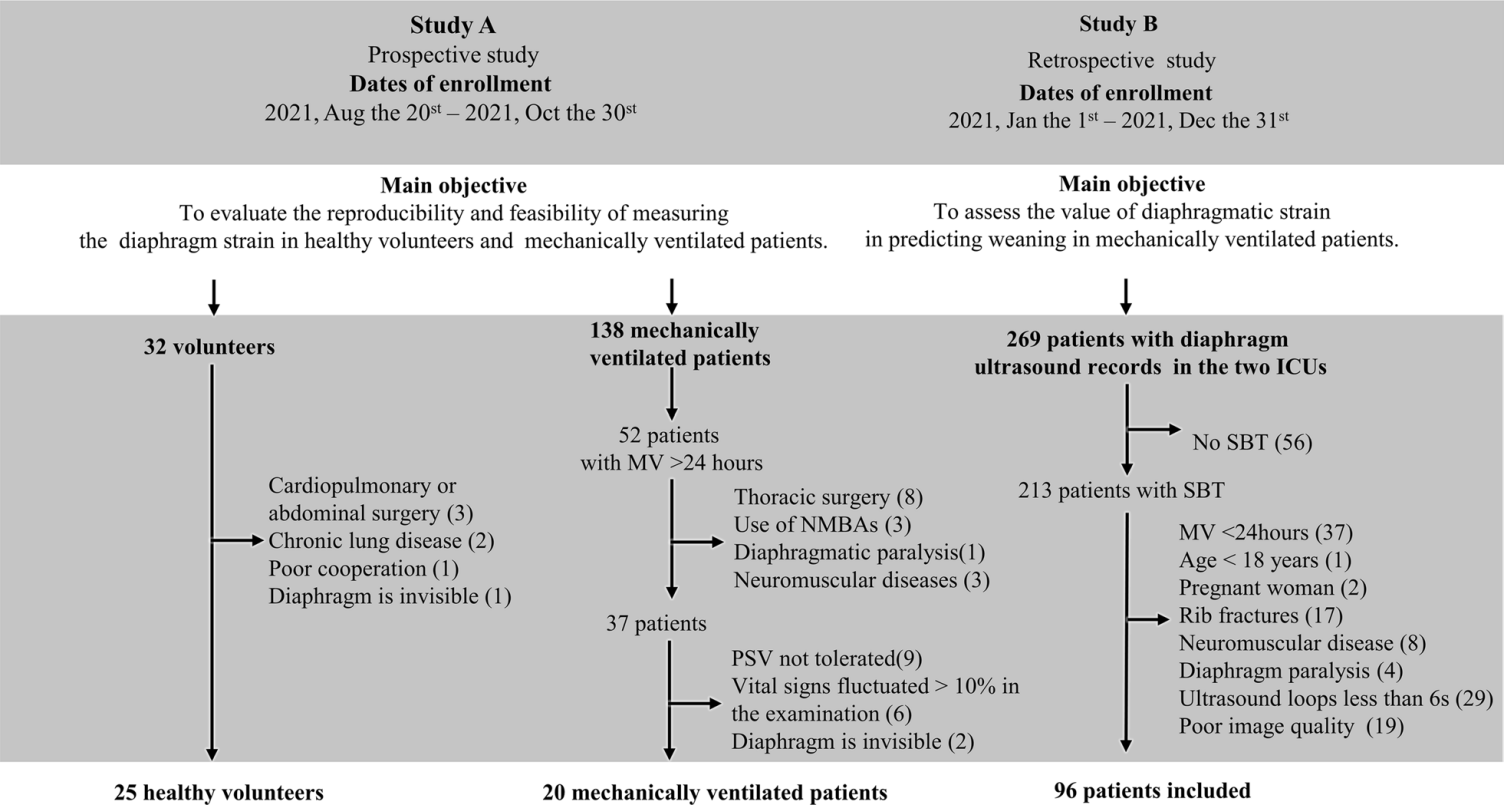
Flow chart of each phase. SBT, spontaneous breathing trial; MV, mechanical ventilation; ICU, intensive care unit; NMBAs, neuromuscular blocking agents; PSV, pressure support ventilation

This study was performed in the intensive care unit (ICU) of two teaching hospitals: Wuhu Hospital, East China Normal University (Wuhu, China) and Zhongnan Hospital of Wuhan University (Wuhan, China). The ICU of Wuhu Hospital has 13 beds, whereas the ICU of Zhongnan Hospital has 66 beds. Both ICUs are managed by full-time ICU doctors and nurses. The main study was divided into two studies (Study A and Study B). The characteristics of each study are depicted in Fig. 1. Protocols involving patients were approved by the Institutional Review Boards of both participating institutions and adhered to all relevant national rules, institutional policies, and the 2008 Helsinki Declaration (2021117 and 202121). Written informed consent was obtained from each patient (or volunteer) or his or her authorized representatives.

### Subjects and study designs

Study A was a prospective observational programme devoted to evaluating the repeatability and value of measuring diaphragmatic strain using 2D speckle tracking technology in healthy subjects and critically ill patients. The study period was from August 20^th^, 2021 to October 30^th^, 2021. Healthy adults over 18 years with normal exercise tolerance met the inclusion criteria (defined as being able to climb three flights of stairs with relative ease). Subjects with a history of prior neuromuscular disease, chronic lung disease (such as moderate to severe restrictive or obstructive pulmonary disease), and cardiopulmonary or abdominal surgery were excluded. Critically ill patients were considered eligible if they had been on invasive mechanical ventilation through endotracheal intubation for more than 24 h and had been on pressure support ventilation (PSV) with spontaneous breathing with pressure less than 15 cmH_2_O for at least 2 h. The decision to initiate PSV was made by the attending physician who was not involved in the study. Invisible right diaphragms with ultrasound, diaphragmatic paralysis, known neuromuscular diseases (such as Guillain‒Barré and myasthenia gravis), thoracic surgery, and poor image quality were all exclusion criteria. Data were excluded if the patient's vital signs (such as respiratory rate and heart rate) fluctuated more than 10% with respiratory distress or if the patients were agitated or sweated during the measurement. Diaphragmatic conventional ultrasound indicators (DE, DTei, DTee, and DE) and maximal DLS during the inspiratory phase of the heathy subjects' eupnoea and deep breathing and critically ill patients (10-min interval) were performed. The volunteers/patients were assessed by two operators (Q.X. and Y.Q.) after a 10-min rest, and interoperator reproducible calculations were performed. The two operators were completely unaware of each other's findings. To allow for reproducible intraoperator calculations, each operator obtained two sets of measurements 10 min apart. Each indicator was measured thrice and averaged (Additional file 1: Fig. S1).

Study B was a retrospective controlled study to assess the value of maximal DLS measured by 2D speckle tracking in predicting weaning in critically ill mechanically ventilated patients. Weaned patients are routinely assessed with diaphragmatic ultrasound, which is typically performed 30 min after a spontaneous breathing test (SBT) or before reventilation. We selected all patients with IMV for more than 24 h with diaphragmatic ultrasound measurements recorded and stored in the ultrasound device (Vivid7, GE, USA and Vivid S6, GE, USA) during their first SBT between January 2021 and December 2021. The SBT criteria met all the indications for SBT (Additional file 2), which have been published elsewhere [14, 25]. Diaphragmatic ultrasound images or videos stored in the ultrasound device include DTF, DE, diaphragmatic thickness, and videos (> 6 s) of diaphragmatic motion measured with a linear probe. The exclusion criteria were as follows: (1) age < 18 years; (2) pregnancy; (3) pneumothorax, mediastinal emphysema, or a history of chest surgery; (4) flail chest or combined rib fractures; (5) neuromuscular disease, (6) poor image quality or ultrasound loops less than 6 s; and (7) diaphragm ultrasonography revealing paradoxical movement of the unilateral diaphragm or history of diaphragmatic paralysis.

Age, sex, vital signs, disease severity score indicators, duration of mechanical ventilation before weaning, conventional diaphragmatic ultrasound parameters (DTF, DE), maximal DLS, and weaning outcomes served as study variables. Tolerating at least 48 h of spontaneous breathing was considered successful weaning from mechanical ventilation, including noninvasive mechanical ventilation and high-flow oxygen therapy, and weaning failure was defined as requiring reintubation within 48 h or death within 7 days after extubation [12, 14, 26].

### Measurements

Images were acquired using a commercial ultrasound machine (Vivid 7, GE, USA and Vivid S6, GE, USA) with a linear array transducer (Vivid S6: 4.0–13.0 MHz; Vivid 7: 4.0–11.0 MHz). The subjects were positioned in a semirecumbent position at 45 degrees, and all diaphragmatic ultrasounds were performed by Q.X. and Y.Q. In Study B, all diaphragmatic ultrasounds performed by specific investigators (Q.X. and Y.Q.) who were trained senior residents or attending physicians with extensive experience in critical care ultrasound were retrospectively selected. A diaphragm ultrasound video and the measurement protocol of conventional diaphragmatic ultrasound indices are provided in the supplemental material (Additional file 2).

### DLS measurement using a 2D speckle-tracking analysis protocol

**Fig. 2:**
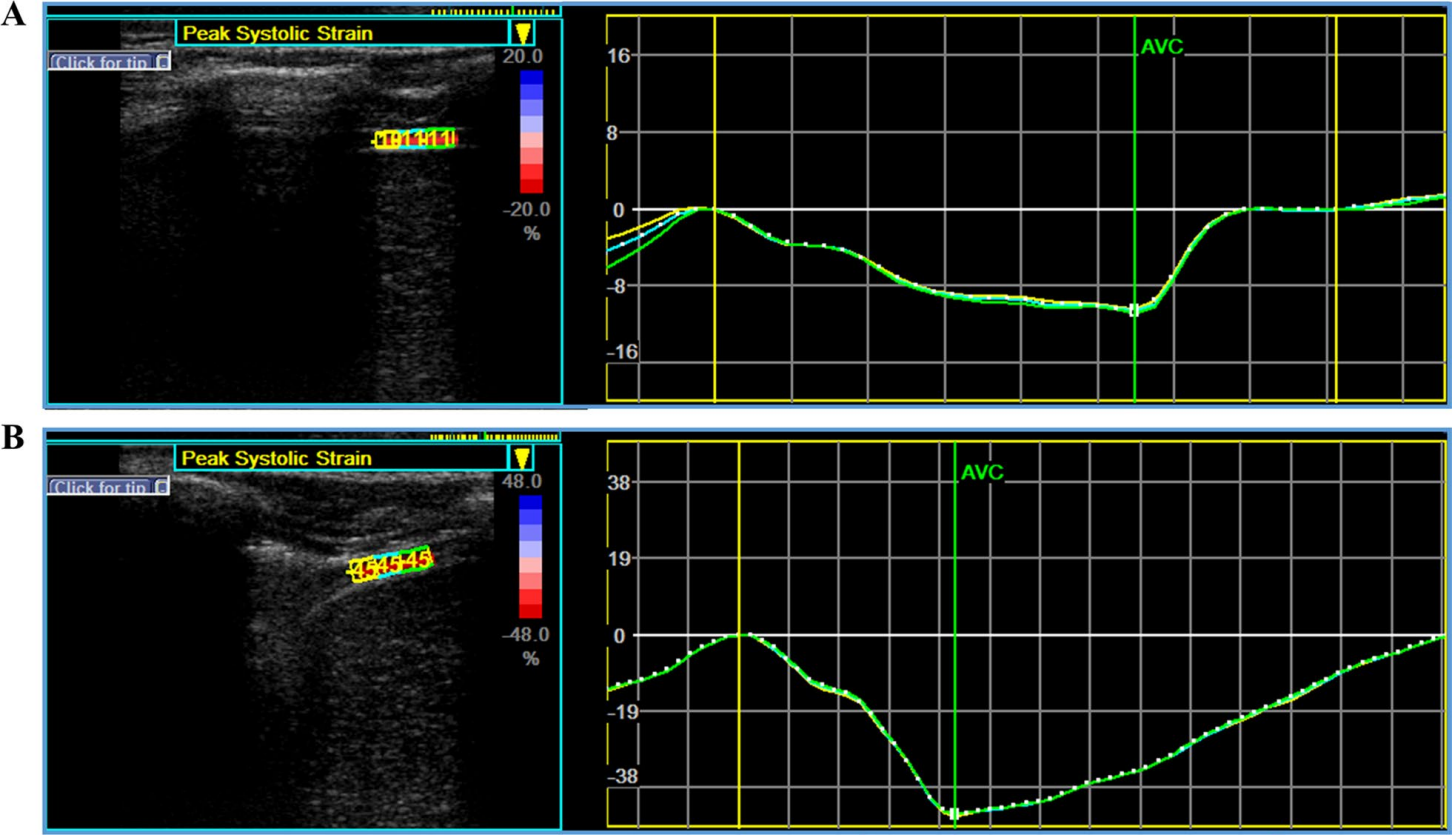
Typical profiles of diaphragm longitudinal strain quantification by sparkle tracking in eupnea and deep breathing. **A:** In eupnea, the maximum diaphragm longitudinal strain value of each segment of the region of interest. Furthermore, the curves represent the strain over time of each segment during diaphragmatic contraction (one respiration cycle). The white squares represent the maximum longitudinal strain values (white arrow). The strain values vary during the time due to diaphragmatic contraction induced by breathing. **B:** Maximal diaphragm longitudinal strain value of each segment of the region of interest in deep breathing. And, the curves are representing strain over time of each segment for diaphragmatic contraction. The white squares represent the maximum longitudinal strain values (white arrow). The strain values change over time as a result of diaphragmatic contraction caused by breathing. In this example, the maximal diaphragm longitudinal strain value in eupnea (**A**) and deep breathing(**B**) was -40% and -21%, respectively. The more negative value means the higher degree of deformation (contraction)

More than 6 s of video of diaphragm movement was collected under these optimum conditions for off-line speckle-tracking analysis using EchoPacs (GE Healthcare, Milwaukee, MI). After selecting the first respiratory cycle (inspiration–expiration) by visual observation of the diaphragmatic contraction-diastole, a speckle tracking filter was used. This filter is analysed using the apical dual-chamber echocardiographic mode; the region of interest (ROI) consists of three diaphragmatic segments (yellow, blue, and green). The upper and lower limits of the ROI represent the pleura and outer peritoneum, respectively. The ROI must be visible throughout the respiratory cycle, and the ROI must be adjusted or redefined multiple times before accurate tracking can be achieved. After a visual assessment, three segments of the diaphragmatic tracking ROI were confirmed. Moving the yellow and green lines on the left (usually corresponding to the shutting of the aortic valve) to assess the maximum longitudinal strain difference between the lowest and highest points of the curve can modify the position at which the analysis cycle begins. The analysis provided strain curves and maximum strain values for each diaphragm segment as well as the mean strain curves, and the maximum longitudinal strain values were recorded for all segments (Fig. 2 and Additional file 3: Video 1).

### Statistical analyses

Continuous variables were summarized as either means and standard deviations or medians and interquartile ranges (IQRs) according to the type of data distribution (normal or skewed). The Kolmogorov‒Smirnov test was used to determine the normality of a variable's distribution. Frequencies and percentages were used to describe categorical variables. Pearson’s correlation coefficient was used to assess the correlation between conventional diaphragmatic ultrasound variables and diaphragmatic strain, and a t test was used for direct comparison. The correlation coefficient was interpreted as follows: 0.00–0.30 indicates a negligible correlation, 0.30–0.50 low, 0.50–0.70 moderate, 070–0.90 high and 0.90–1.00 very high [27]. Intraclass correlation coefficients (ICCs) and Bland‒Altman plots were used to analyse the intra- and interoperator reliability of the DLS. ICCs are classified as indicating poor, moderate, good or excellent reliability [28]. The DLS differences between sexes were compared using the Mann‒Whitney test. Based on previously published research, the sample size for this exploratory investigation was predicted to be 25 healthy volunteers and 20 critically ill mechanically ventilated patients [29, 30]. For Study B, receiver operating characteristic curves (ROC) were used to evaluate the performance of predicting weaning outcome. The optimal threshold for each index was determined as the value associated with the best Youden index. A nonparametric method was used to compare the area under the receiver operating characteristic curve (AUC) of different weaning parameters [31]. There was no data loss or follow-up loss. In all cases, *P* values < 0.05 were considered statistically significant, and a two-tailed test was used to test the hypothesis. All statistics were performed using SPSS 22.0 for Windows (IBM Corporation, Armonk, NY, USA) and MedCalc Windows (MedCalc Software bvba 13, Ostend, Belgium).

## Results

### The feasibility for DLS in study A

Thirty-two volunteers and thirty-seven mechanical ventilated patients were assessed for eligibility in Study A. Twenty-four patients were excluded (Fig. 1). Patients’ baseline characteristics are summarized in Tables 1 and 2. Our protocol specifies that 3 ultrasound cycleloops will be acquired for each measurement. In total, 840 loops were obtained upon completion of cineloop acquisition (Figure S1). However, 93 cineloops were excluded. In 56 cineloops, the diaphragm was covered by the lungs throughout the respiratory cycle, 29 cineloops were excluded due to insufficient tracking of the ROI throughout the respiratory cycle, and 8 cineloops were incomplete. Therefore, analysis was ultimately possible in 88.9% (747/840) of ultrasound cineloops.

**Table 1:** Characteristics and in healthy subjects

Characters	Results (n = 25)
Age (years)	48.92 ± 16.29
Male (%)	14 (70%)
Height (cm)	170.2 ± 8.32
Weight (kg)	63.36 ± 10.65
Body mass index (kg/m^2^)	21.79 ± 2.79
Hypertension (%)	3 (12.0)
Diabetes (%)	1 (4.0)
Chronic pulmonary disease (%)	2 (8.0)

**Table 2:** Critical ill mechanical ventilator patient characters

Characters	Results (n = 20)
*Baseline*	
Age (years)	57.40 ± 16.43
Male (%)	14 (70%)
APACHE II score	15.0 ± 4.73
Temperature (℃)	37.01 ± 0.64
Heart rate (bpm)	97.35 ± 17.79
Respiratory rate (bpm)	17 [16–19]
Systolic pressure (mmHg)	127.4 ± 19.86
Diastolic pressure (mmHg)	70.45 ± 8.67
Support pressure (cmH_2_O)	15.50 [14.75—17.00]
Positive end expiratory pressure (cmH_2_O)	5.50 [5.00—6.25]
PaO_2_ / FiO_2_ ratio (mmHg)	262.00 [179.50—328.15]
*Comorbidity*	
Hypertension (%)	4 (20.0)
Diabetes (%)	2 (10.0)
Chronic pulmonary disease (%)	3 (15.0)
Malignant solid tumor (%)	1 (5.0)
Chronic kidney disease (%)	2 (10.0)
Others (%)	2 (10.0)
*Etiology of mechanical ventilation*	
Postsurgical respiratory failure	3 (15.0)
Sepsis	7 (35.0)
ARDS	4 (20.0)
AECOPD	2 (10.0)
Trauma	1 (5.0)
Others	3 (15.0)

Data are presented as median with interquartile range or mean ± standard deviation (SD) or frequencies with percentages. *APACHE II* Acute Physiology, Age, Chronic Health Evaluation II; *PaO*_*2*_ Partial arterial oxygen pressure; *FiO*_*2*_ Fraction of inspired oxygen; *ARDS* Acute Respiratory Distress Syndrome; *AECOPD* Acute exacerbation of chronic obstructive pulmonary disease; *PaCO*_*2*_ Arterial partial pressure of carbon dioxide

#### DLS in study A

**Fig. 3:**
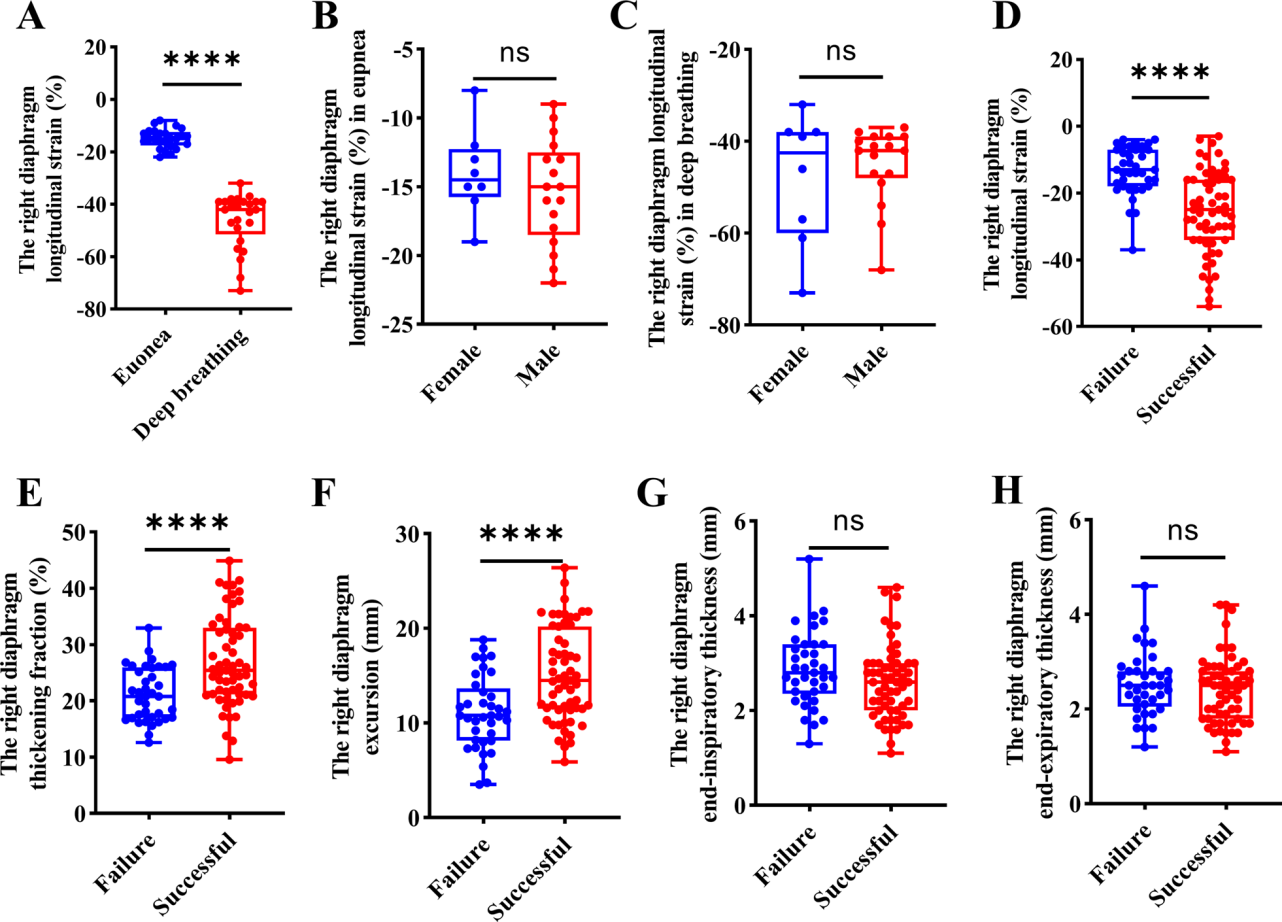
Box plots of maximal diaphragm longitudinal strain value (**A**) and gender difference in healthy volunteers during eupnea (**B**) or deep breathing (**C**). Maximum diaphragm longitudinal strain box plots (**D**), diaphragm thickening fraction (**E**), diaphragm excursion (**F**), diaphragmatic thickness at end-inspiration (**G**), diaphragmatic thickness at end-expiration (**H**) in weaning success and failure groups

The DLS (%) values in the diaphragmatic zone of apposition in *healthy subjects (in* eupnoea and deep breathing) and mechanically ventilated patients were −15 (12.75 to 17.25), −42.0 (−35.0 to 50.25), and −16 (−10.5 to 34.0), respectively. No differences in sex were noted between *healthy subjects* and mechanically ventilated patients (*P* > 0.05) (Fig. 3A–C).

#### ICC for DLS in study A

**Fig. 4:**
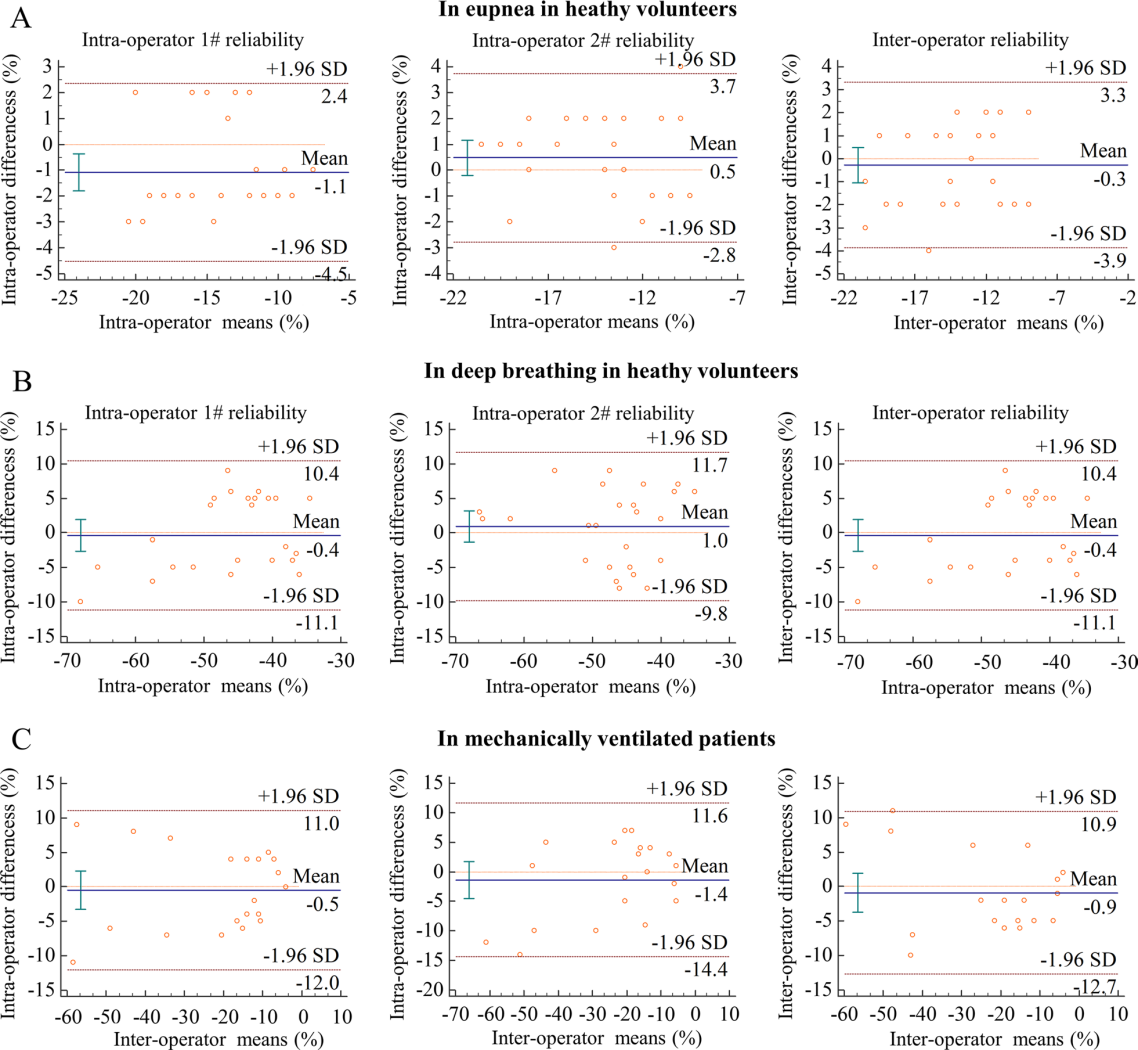
Bland–Altman plot of the difference in maximal diaphragm longitudinal strain value between operators in healthy subjects and critical ill mechanical ventilation patients. Under eupnea (**A**) and deep breathing (**B**) in healthy subjects, and in critical ill mechanical ventilation patients (**C**)

*In healthy volunteers with eupnoea*, intraoperator reliability was good in the first operator with an ICC of 0.86 (95% CI 0.63; 0.95) and good in the second operator with an ICC of 0.87 (95% CI 0.74; 0.94]. Interoperator reliability was good, with an ICC of 0.87 (95% CI 0.73; 0.94) (Fig. 4A, Additional file 1: Table S1). During deep breathing, the intraoperator reliability of the first and second operators was good with ICCs of 0.84 (95% CI 0.66; 0.92) and 0.80 (95% CI 0.59; 0.90), respectively. Interoperator reliability was good with an ICC of 0.78 (95% CI 0.57; 0.90) (Fig. 4B, Additional file 1: Table S1). In *mechanically ventilated patients,* intraoperator reliability was excellent in the first and second operators with ICCs of 0.95 (95% CI 0.87; 0.98) and 0.92 (95% CI 0.82; 0.97), respectively. Interoperator reliability was excellent with an ICC of 0.94 (95% CI 0.85; 0.97) (Fig. 4C, Additional file 1: Table S1).

#### DLS and weaning parameters in study B

A total of 269 patients with diaphragmatic ultrasound records were chosen, and 96 of these patients participated in Study B (Fig. 2). Patient characteristics are presented in Table 3. Among the 96 patients, 59 (61.46%) patients successfully weaned from mechanical ventilation, and the remaining 37 (38.54%) patients failed to be weaned from mechanical ventilation. Compared to the successfully weaned group, the 28-day mortality of patients who failed to be weaned was significantly higher (43.2% vs. 13.6%, *P* = 0.002), and the percentage of high-flow nasal cannula administration (43.2% vs. 16.9%, *P* = 0.01) was significantly higher. No significant difference in noninvasive positive pressure ventilation administration (21.6% vs. 8.5%, *P* = 0.127) was noted between the success and failure groups (Table 3).

**Table 3:** Demographics, laboratory test, type of ICU admission, comorbidity, etiology of mechanical ventilation, and treatment in the examined patients

Characters	Success group (n = 59)	Failure group (n =37)	*p*-Value
*Baseline*			
Age (years)	64.00 [54.50–74.00]	67.00 [58.00–77.00]	0.515
Male (%)	35 (59.3)	25 (67.6)	0.551
APACHE II score	20.00 [18.00–25.00]	18.00 [13.00–21.00]	0.002
Temperature (℃)	37.00 [36.65–37.30]	37.00 [36.60–37.30]	0.979
Heart rate (bpm)	96 [83–112]	96 [82–115]	0.827
Respiratory rate (bpm)	18 [16–19]	22 [17–26]	0.001
Systolic pressure (mmHg)	131 [123–141]	130 [117–139]	0.331
Diastolic pressure (mmHg)	69 [64–72]	69 [65–71]	0.656
PaO_2_ / FiO_2_ ratio (mmHg)	252.50 [212.85–280.70]	241.40 [216.70–280.20]	0.665
Support pressure on SBT (cmH_2_O)	7.00 [6.00–8.00]	7.00 [6.00–8.00]	0.83
Positive end expiratory pressure (cmH_2_O)	5.00 [5.00–5.00]	5.00 [5.00–5.00]	0.76
*Ventilator days before SBT (days)*	4.00 [3.00–5.50]	8.00 [6.00–9.00]	< 0.001
*Type of ICU admission (%)*			0.988
Medical	29 (49.2)	18 (48.6)	
Elective surgical	18 (30.5)	11 (29.7)	
Urgent surgical	12 (20.3)	8 (21.6)	
*Comorbidity*			
Hypertension (%)	19(32.2%)	12 (32.4%)	> 0.999
Diabetes (%)	9 (15.3)	6 (16.2)	> 0.999
Chronic pulmonary disease (%)	8 (13.6)	5 (13.5)	> 0.999
Chronic heart failure (%)	7 (11.9)	4 (10.8)	> 0.999
Malignant solid tumor (%)	7 (11.9)	4 (10.8)	> 0.999
Chronic kidney disease (%)	6 (10.2)	3 (8.1)	> 0.999
Others (%)	4 (6.8)	2 (5.4)	> 0.999
*Etiology of mechanical ventilation*			> 0.999
Postsurgical respiratory failure	8 (13.6)	4 (10.8)	
Sepsis	18 (30.5)	11 (29.7)	
ARDS	9 (15.3)	6 (16.2)	
AECOPD	9 (15.3)	6 (16.2)	
Acute left heart failure	6 (10.2)	4 (10.8)	
Trauma	2 (3.4)	1 (2.7)	
Others	8 (13.6)	4 (10.8)	
*Outcomes*			
Failed SBT	–	30 (81.1)	NA
Reintubation in 48 h	–	4 (10.8)	NA
Death within 7 days of extubation	–	3 (8.1)	NA
NPPV	5 (8.5)	8 (21.6)	0.127
High-flow oxygen therapy	10 (16.9)	16 (43.2)	0.01
28-day mortality	8 (13.6)	16 (43.2)	0.002

Data are presented as median with interquartile range or frequencies with percentages. *APACHE II* Acute Physiology, Age, Chronic Health Evaluation II; *PaO*_*2*_ Partial arterial oxygen pressure; *FiO*_*2*_ Fraction of inspired oxygen; *ARDS* Acute Respiratory Distress Syndrome; *AECOPD* Acute exacerbation of chronic obstructive pulmonary disease; *NMBAs* Neuromuscular blocking agents; *SBT* Spontaneous breathing trial; *NPPV* Non-invasive positive pressure ventilation

DLS (%), DTF (%), and DE displayed statistically significant differences between the success and failure groups: DLS (%) [success group –25.00 (−34 to −16), failure group –13.00 (−18.00 to −7.00); *P* < 0.001], DTF (%) [success group 25.40 (21.18 to 32.84), failure group 20.82 (17.05 to 25.86); *P* < 0.001], and DE (mm) [success group 14.50 (11.55 to 19.50), failure 10.80 (8.20 to 13.30); *P* < 0.001]. In contrast, DTee (mm) and DTei (mm) were not significantly different between groups [success group 2.80 (2.20 to 3.40), failure group 2.70 (2.40 to 3.20); *P* = 0.827] and [success group 3.60 (2.80 to 4.10), failure group 3.30 (2.80 to 3.90); *P* = 0.564], respectively (Fig. 3DE–H).

**Fig. 5:**
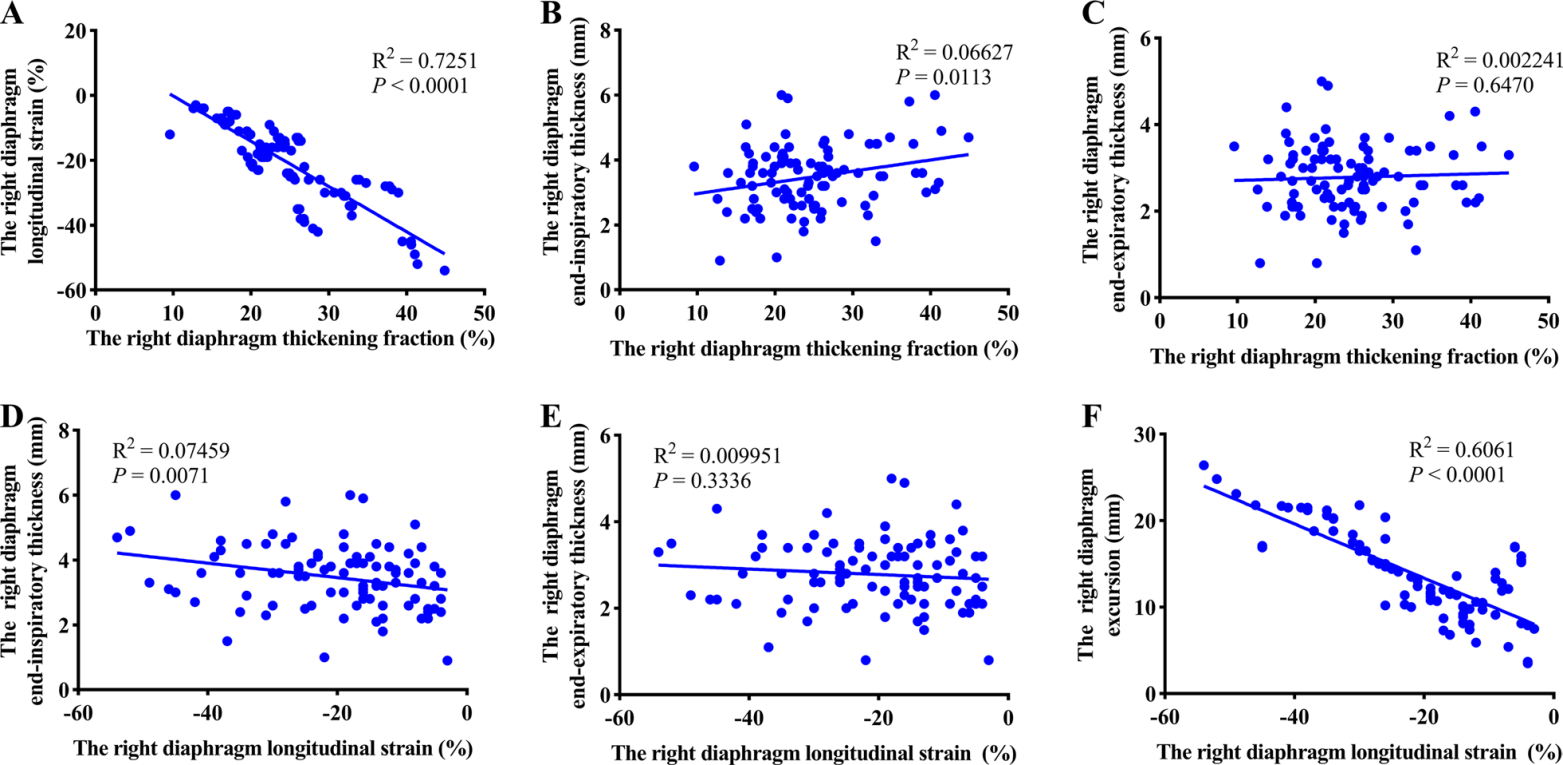
Graphical representation of correlation among various diaphragm assessment methods. **A** Maximal diaphragm longitudinal strain vs. diaphragm thickening fraction (R^2^ = 0.73, *P* < 0.0001), **B** Diaphragm thickening fraction vs. diaphragmatic thickness at end inspiration (R^2^ = 0.07, *P* < 0.0113), **C** Diaphragm thickening fraction vs. diaphragmatic thickness at end expiration (R^2^ = 0.002, *P* = 0.647), **D** Maximal diaphragm longitudinal strain vs. diaphragmatic thickness at end inspiration (R^2^ = 0.07, *P* = 0.0071), **E** Maximal diaphragm longitudinal strain vs. diaphragmatic thickness at end expiration (R^2^ = 0.01, *P* = 0.3336), **F** Maximal diaphragm longitudinal strain vs. diaphragm excursion (R^2^ = 0.61, *P* < 0.0001)

DLS demonstrated a fair linear relationship with DTF (Pearson R^2^ = 0.73, *P* < 0.0001) and DE (Pearson R^2^ = 0.61, *P* < 0.0001). However, a weak or no relationship existed between DLS and diaphragmatic thickness (DTee, R^2^ = 0.01, *P* = 0.3336; DTei, R^2^ = 0.07, *P* = 0.0071). Similarly, a weak or no correlation was noted between DTF and diaphragmatic thickness (DTee, R^2^ = 0.002, *P* = 0.647; DTei, R^2^ = 0.07, *P* = 0.0113) (Fig. 5).

**Fig. 6:**
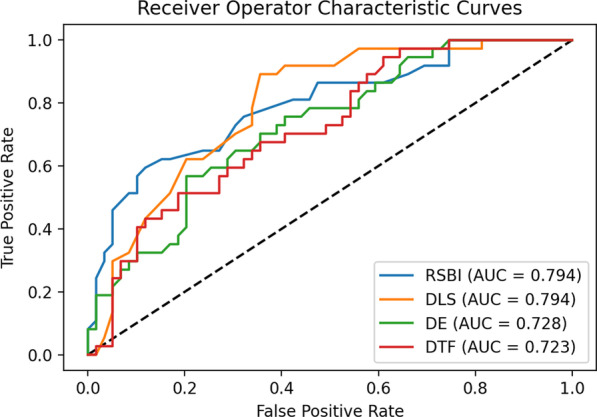
ROC curves of RSBI (blue line), DE (green line), DTF (red line), and DLS (orange line) for the prediction of successful weaning. RSBI: Rapid shallow breathing index (the ratio of respiratory frequency to tidal volume); DLS, maximal diaphragm longitudinal strain; DTF, diaphragm thickening fraction; DE, diaphragm excursion

The AUCs of DLS, RBSI, DTF and DE for the prediction of successful weaning were 0.794, 0.794, 0.0.723 and 0.728, respectively. The best cut-off values of DLS, DTF, and DE for predicting weaning success were less than -21% (sensitivity 89.19%, specificity 64.41%), greater than 83 (sensitivity 59.46%, specificity 88.14%), greater than 27.39% (sensitivity 94.59%, specificity 38.98%), and greater than 11.2 (sensitivity 56.76%, specificity 79.66%), respectively (Fig. 6). DLS exhibits a high predictive value for mechanical weaning. However, DLS offers no advantage over RSBI (Table 4).

**Table 4:** Comparison of AUC-ROC curves of the four indices to predict spontaneous breathing trial failure

Characters	Z	*P*-value
*Maximum diaphragm longitudinal strain vs.*		
Diaphragm thickening fraction	2.09	**0.0368**
Diaphragm excursion	1.31	0.1889
Rapid shallow breathing index	0.00	> 0.9999
*Diaphragm thickening fraction vs.*		
Diaphragm excursion	0.07	0.9421
Rapid shallow breathing index	1.06	0.2881
*Diaphragm excursion vs.*		
Rapid shallow breathing index	0.85	0.3959

*AUC* Area under the receiver operating characteristic curve

## Discussion

To our knowledge, this is the first study to focus on the ability of DLS to predict weaning outcome in patients with mechanical ventilation. The results demonstrated that DLS evaluation using 2D speckle tracking with a linear array transducer was feasible and reproducible, and a DLS of less than -21% had good performance for predicting successful weaning (AUC = 0.794).

At present, 2D measurements, such as DTF, DE, and diaphragmatic thickness, are widely used to assess diaphragmatic function. However, the accuracy of 2D measurements in predicting successful weaning is highly controversial [14, 32–34]. For instance, 2D measurements can only assess the deformation of the diaphragmatic musculature in the transverse direction; longitudinal measures of the contraction direction cannot be obtained. Several studies have been conducted to explore speckle tracking as a promising approach to evaluating diaphragmatic contractility in healthy subjects [15, 18, 29, 35, 36]; however, few studies have reported the use of speckle tracking to assess diaphragmatic contractility among mechanically ventilated patients. Fritsch and colleagues reported the feasibility of diaphragmatic speckle tracking in a cohort of 20 patients after an aortocoronary bypass graft procedure [29]. This finding was in accordance with our study. We found that speckle tracking ultrasonography has high feasibility and repeatability in both healthy subjects and critically ill patients on mechanical vent elation, which further strengthens the evidence for the implementation of speckle tracking, especially for patients on mechanical ventilation.

In addition to the feasibility and repeatability, the ability of diaphragmatic speckle tracking technology to reflect diaphragmatic contraction function should also be considered. Oppersma and colleagues used diaphragmatic electric activity and transdiaphragmatic pressures to compare the ability of different diaphragmatic ultrasound technologies to estimate diaphragmatic contraction and found that speckle tracking is superior to conventional parameters [36]. Fritsch and colleagues also suggested that speckle tracking ultrasonography was able to depict alterations in diaphragmatic function better than conventional ultrasonographic measurements [29]. In our research, significant differences in DLS values were noted patients with eupnoea, deep inspiration, and mechanical ventilation, and these differences were consistent with the physiological and pathophysiological changes in the diaphragm. These findings also indicated that speckle tracking ultrasonography could effectively reflect diaphragmatic contraction function.

The accuracy of ultrasonography may be affected by many factors, such as the skills and experience of operators, the incident angle of the ultrasound beam, and imaging quality. The diaphragm is a thin muscle with an average thickness of only 1.7–2 mm (mm) in healthy subjects when measured in the zone of apposition [37], and the diaphragm may be even thinner during mechanical ventilation [11, 32, 38]. Therefore, it is difficult to accurately measure the thickness of the diaphragm using ultrasound [30]. Goligher and colleagues confirmed that great error existed in the measurement of diaphragmatic ultrasound data [32]. For example, DTF is often used to reflect diaphragmatic function, but the accuracy of this methodology is controversial. The inaccuracy is more pronounced when the boundaries between the visceral pleura and peritoneum and diaphragm fibres are not well defined. This condition is particularly common in patients with diaphragmatic atrophy, which is often associated with mechanical ventilation of critically ill patients [26, 38, 39]. On the other hand, DTF in healthy individuals ranged from 24.5 to 53.2% during normal breathing and was as high as 131% during forced inspiration; this large-scale fluctuation also increases the difficulty in obtaining accurate measurements. A recent study showed that speckle tracking technology can effectively measure tissue strain, which can be accurately measured to 1% [23]. Our study also showed that speckle tracking quantified diaphragmatic strain with good to excellent intra- and interoperator ICC under conditions of eupnoea, deep breathing, and mechanical ventilation, suggesting that the speckle tracking technique exhibits high accuracy in quantifying DLS.

As of January 2022, hundreds of studies have been conducted to determine whether diaphragmatic ultrasound predicts successful weaning. However, the main parameters, such as RSBI, DTF, DE, and diaphragmatic thickness, were not ideal for predicting successful weaning, and even high-quality multicentre studies have shown that these parameters cannot predict outcome after weaning [8, 9, 14]. In this study, we found that diaphragmatic strain represents an important indicator for assessing diaphragmatic contraction function. However, compared to RSBI, it is not more effective in predicting weaning success potentially because diaphragmatic dysfunction is merely one of several factors influencing weaning success. Furthermore, there are other causes of diaphragmatic dysfunction, including intrinsic muscle weakness, residual sedation, or simply a lack of necessary ventilatory effort [14, 35]. Therefore, no effective parameter or method, including diaphragmatic ultrasound, is currently available for the accurate prediction of weaning [14, 33]. It is hypothesized that it will be difficult to obtain high sensitivity and specificity for any diaphragmatic ultrasound index in future studies.

There are several limitations to our study. First, we only analysed the right diaphragm to reduce volunteer pressure and effort. Second, no invasive gold standard measurements, such as transdiaphramatical pressure or diaphragm electrical activity, were performed. However, a recent study found a connection between these parameters and speckle tracking [36]. Third, current speckle tracking techniques, which are primarily used for myocardial analyses, may experience difficulties in accurately tracking each specific greyscale pixel (known as ‘kernels’) possibly due to differences in muscle echoes. Therefore, the ROI must be adjusted or redefined multiple times before accurate tracking can be achieved. This issue was also noted in a prior study [18]. As a result, it is critical to create or update software that is specifically designed for analysing diaphragmatic tissue. As an added feature, an online analysis tool can increase the method's usability. Fourth, our initial aim was to explore whether speckle tracking ultrasonography could accurately assess diaphragm function. However, before the research started, Fritsch SJ et al. published similar research, demonstrating that speckle tracking ultrasonography depicts alterations in diaphragmatic function after surgery better than 2D ultrasonographic measurements [29]. Therefore, we modified the protocol. In mechanically ventilated patients, only the feasibility and reliability of the use of speckle tracking to measure the longitudinal strain of the diaphragm was evaluated. Fifth, transverse strain is worth measuring. However, analysis using the apical dual-chamber echocardiographic mode was unable to measure lateral and radial strain due to diaphragm thickness, and an ROI of at least 1–2 cm was required for analysis. However, with the advancement of technology, transverse strain of the diaphragm may represent another direction of our future research.

## Conclusions

Diaphragmatic longitudinal strain quantification using speckle tracking is easily achievable in both healthy subjects and mechanically ventilated patients and has a high predictive value for weaning success. However, it has no advantage over RSBI. Future research should assess its value as a predictor of weaning.

## Supplementary Information

**Additional file 1**: **Figure S1** Intra- and inter-operator reliability analysis protocol. **Table S1** Intra- and inter-operator reliability analysis and sample t-test.

**Additional file 2**. SBT indications and obtaining the diaphragm ultrasound video and measurement protocol.

**Additional file 3**: **Video 1** Video clip of the positioning of the region of interest for speckle tracking analysis of diaphragm contraction..

## Data Availability

All data generated or analyzed during this study are included in this published article.

## Abbreviations

DLS: Diaphragmatic longitudinal strain
DE: Diaphragmatic excursion
DTF: Diaphragmatic thickening fraction
DTei: Diaphragmatic thickness at end-inspiration
DTee: Diaphragmatic thickness at end-expiration
ICU: Intensive care unit
ICC: Intraclass correlation coefficient
IQR: Interquartile ranges
MIP: Maximum inspiratory pressure
mm: Millimeters
NMBAs: Neuromuscular blocking agents
PSV: Pressure support ventilation
ROC: Receiver operating characteristic
ROI: Region of interest
RSBI: Rapid shallow breathing index
SBT: Spontaneous breathing trials
2D: Two-dimensional

## References

[1] Esteban A, Anzueto A, Frutos F (2002). Characteristics and outcomes in adult patients receiving mechanical ventilation: a 28-day international study. JAMA.

[2] Funk GC, Anders S, Breyer MK (2010). Incidence and outcome of weaning from mechanical ventilation according to new categories. Eur Respir J.

[3] Cislaghi F, Condemi AM, Corona A (2009). Predictors of prolonged mechanical ventilation in a cohort of 5123 cardiac surgical patients. Eur J Anaesthesiol.

[4] Suarez-Pierre A, Fraser CD, Zhou X (2019). Predictors of operative mortality among cardiac surgery patients with prolonged ventilation. J Card Surg.

[5] Hudson MB, Smuder AJ, Nelson WB, Bruells CS, Levine S, Powers SK (2012). Both high level pressure support ventilation and controlled mechanical ventilation induce diaphragm dysfunction and atrophy. Crit Care Med.

[6] Nelson JE, Cox CE, Hope AA, Carson SS (2010). Chronic critical illness. Am J Respir Crit Care Med.

[7] Thille AW, Harrois A, Schortgen F, Brun-Buisson C, Brochard L (2011). Outcomes of extubation failure in medical intensive care unit patients. Crit Care Med.

[8] Baptistella AR, Sarmento FJ, da Silva KR (2018). Predictive factors of weaning from mechanical ventilation and extubation outcome: a systematic review. J Crit Care.

[9] Trivedi V, Chaudhuri D, Jinah R (2022). The usefulness of the rapid shallow breathing index in predicting successful extubation: a systematic review and meta-analysis. Chest.

[10] Boussuges A, Gole Y, Blanc P (2009). Diaphragmatic motion studied by m-mode ultrasonography: methods, reproducibility, and normal values. Chest.

[11] Zambon M, Greco M, Bocchino S, Cabrini L, Beccaria PF, Zangrillo A (2017). Assessment of diaphragmatic dysfunction in the critically ill patient with ultrasound: a systematic review. Intensive Care Med.

[12] Ferrari G, De Filippi G, Elia F, Panero F, Volpicelli G, Apra F (2014). Diaphragm ultrasound as a new index of discontinuation from mechanical ventilation. Crit Ultrasound J.

[13] Alam MJ, Roy S, Iktidar MA (2022). Diaphragm ultrasound as a better predictor of successful extubation from mechanical ventilation than rapid shallow breathing index. Acute Crit Care.

[14] Vivier E, Muller M, Putegnat JB (2019). Inability of diaphragm ultrasound to predict extubation failure: a multicenter study. Chest.

[15] Hatam N, Goetzenich A, Rossaint R (2014). A novel application for assessing diaphragmatic function by ultrasonic deformation analysis in noninvasively ventilated healthy young adults. Ultraschall Med.

[16] Singh A, Voss WB, Lentz RW, Thomas JD, Akhter N (2019). The diagnostic and prognostic value of echocardiographic strain. JAMA Cardiol.

[17] Orde SR, Boon AJ, Firth DG, Villarraga HR, Sekiguchi H (2016). Diaphragm assessment by two dimensional speckle tracking imaging in normal subjects. BMC Anesthesiol.

[18] Ye X, Xiao H, Bai W, Liang Y, Chen M, Zhang S (2013). Two-dimensional strain ultrasound speckle tracking as a novel approach for the evaluation of right hemidiaphragmatic longitudinal deformation. Exp Ther Med.

[19] Frich LH, Lambertsen KL, Hjarbaek J, Dahl JS, Holsgaard-Larsen A (2019). Musculoskeletal application and validation of speckle-tracking ultrasonography. BMC Musculoskelet Disord.

[20] Peterson G, Leary SO, Nilsson D (2019). Ultrasound imaging of dorsal neck muscles with speckle tracking analyses - the relationship between muscle deformation and force. Sci Rep.

[21] Duclos G, Bobbia X, Markarian T (2019). Speckle tracking quantification of lung sliding for the diagnosis of pneumothorax: a multicentric observational study. Intensive Care Med.

[22] Fissore E, Zieleskiewicz L, Markarian T (2021). Pneumothorax diagnosis with lung sliding quantification by speckle tracking: a prospective multicentric observational study. Am J Emerg Med.

[23] Couppe C, Svensson RB, Josefsen CO, Kjeldgaard E, Magnusson SP (2020). Ultrasound speckle tracking of Achilles tendon in individuals with unilateral tendinopathy: a pilot study. Eur J Appl Physiol.

[24] Schrier V, Evers S, Bosch JG, Selles RW, Amadio PC (2019). Reliability of ultrasound speckle tracking with singular value decomposition for quantifying displacement in the carpal tunnel. J Biomech.

[25] Dres M, Dube BP, Goligher E (2020). Usefulness of parasternal intercostal muscle ultrasound during weaning from mechanical ventilation. Anesthesiology.

[26] Theerawit P, Eksombatchai D, Sutherasan Y, Suwatanapongched T, Kiatboonsri C, Kiatboonsri S (2018). Diaphragmatic parameters by ultrasonography for predicting weaning outcomes. BMC Pulm Med.

[27] Mukaka MM (2012). Statistics corner: a guide to appropriate use of correlation coefficient in medical research. Malawi Med J.

[28] Koo TK, Li MY (2016). A guideline of selecting and reporting intraclass correlation coefficients for reliability research. J Chiropr Med.

[29] Fritsch SJ, Hatam N, Goetzenich A (2021). Speckle tracking ultrasonography as a new tool to assess diaphragmatic function: a feasibility study. Ultrasonography.

[30] Goligher EC, Laghi F, Detsky ME (2015). Measuring diaphragm thickness with ultrasound in mechanically ventilated patients: feasibility, reproducibility and validity. Intensive Care Med.

[31] DeLong ER, DeLong DM, Clarke-Pearson DL (1988). Comparing the areas under two or more correlated receiver operating characteristic curves: a nonparametric approach. Biometrics.

[32] Goligher EC, Fan E, Herridge MS (2015). Evolution of diaphragm thickness during mechanical ventilation. Impact of inspiratory effort. Am J Respir Crit Care Med..

[33] Haaksma M, Tuinman PR, Heunks L (2017). Ultrasound to assess diaphragmatic function in the critically ill-a critical perspective. Ann Transl Med.

[34] Goutman SA, Hamilton JD, Swihart B, Foerster B, Feldman EL, Rubin JM (2017). Speckle tracking as a method to measure hemidiaphragm excursion. Muscle Nerve.

[35] Vivier E, Roche-Campo F, Brochard L, Mekontso DA (2017). Determinants of diaphragm thickening fraction during mechanical ventilation: an ancillary study of a randomised trial. Eur Respir J.

[36] Oppersma E, Hatam N, Doorduin J (2017). Functional assessment of the diaphragm by speckle tracking ultrasound during inspiratory loading. J Appl Physiol.

[37] Carrillo-Esper R, Perez-Calatayud AA, Arch-Tirado E (2016). Standardization of sonographic diaphragm thickness evaluations in healthy volunteers. Respir Care.

[38] Supinski GS, Morris PE, Dhar S, Callahan LA (2018). Diaphragm dysfunction in critical illness. Chest.

[39] Dres M, Goligher EC, Heunks LMA, Brochard LJ (2017). Critical illness-associated diaphragm weakness. Intensive Care Med.

