# Supplementary material for: Comparison of assessment of diaphragm function using speckle tracking between patients with successful and failed weaning: a multicentre, observational, pilot study
Source: BMC Pulm Med. 2022 Dec 1;22:459. doi: 10.1186/s12890-022-02260-z (PMC9716762; doi:10.1186/s12890-022-02260-z)
Supplement: Supplementary file 1 — Additional file 1: Figure S1 Intra- and inter-operator reliability analysis protocol. Table S1 Intra- and inter-operator reliability analysis and sample t-test. [file 12890_2022_2260_MOESM1_ESM.docx]

**Supplementary figure and table**

| 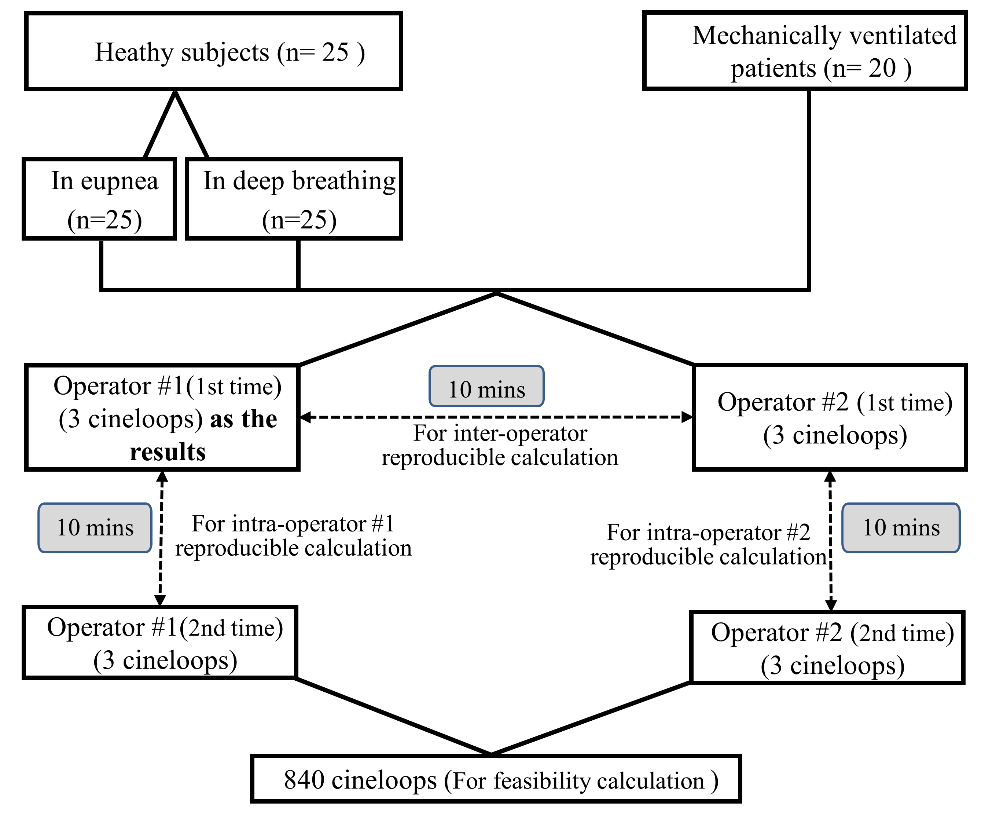  **Figure S1 Intra- and inter-operator reliability analysis protocol** |
| --- |

**Table S1. Intra- and inter-operator reliability analysis and sample t-test.**

| **Characters** | ***ICC*** | ***Mean differences*** | ***Standard***  ***deviation*** | ***95% CI*** | ***Test statistic t*** | ***P*** |
| --- | --- | --- | --- | --- | --- | --- |
| **In eupnea in heathy subjects** |  |  |  |  |  |  |
| Intra-operator 1# | 0.86, 95%CI (0.63 to 0.95) | 0.52 | 2.00 | -0.31 to 1.35 | 1.30 | 0.2065 |
| Intra-operator 2# | 0.87, 95%CI (0.74 to 0.94) | -0.48 | 1.66 | -1.17 to 0.21 | 1.44 | 0.1615 |
| Inter-operator | 0.87, 95%CI (0.73 to 0.94) | 0.28 | 1.84 | -0.48 to 1.04 | 0.76 | 0.4536 |
| **In deep breathing in heathy subjects** |  |  |  |  |  |  |
| Intra-operator 1# | 0.84, 95%CI (0.66 to 0.92) | 0.36 | 5.50 | -1.91 to 2.63 | 0.33 | 0.7463 |
| Intra-operator 2# | 0.80, 95%CI (0.59 to 0.90) | -0.96 | 5.48 | -3.22 to 1.30 | 0.88 | 0.3898 |
| Inter-operator | 0.78, 95%CI (0.57 to 0.90) | -0.72 | 6.25 | -3.30 to 1.86 | 0.58 | 0.5699 |
| **In critically ill patients** |  |  |  |  |  |  |
| Intra-operator 1# | 0.95, 95%CI (0.87to 0.98) | 0.50 | 5.89 | -2.26 to 3.26 | 0.38 | 0.7084 |
| Intra-operator 2# | 0.92, 95%CI (0.82 to 0.97) | -1.4 | 6.63 | -4.51 to 1.71 | 0.94 | 0.3573 |
| Inter-operator | 0.94, 95%CI (0.85 to 0.97) | -0.90 | 6.01 | -3.71 to 1.91 | 0.67 | 0.5110 |
