# Supplementary material for: Comparison of assessment of diaphragm function using speckle tracking between patients with successful and failed weaning: a multicentre, observational, pilot study
Source: BMC Pulm Med. 2022 Dec 1;22:459. doi: 10.1186/s12890-022-02260-z (PMC9716762; doi:10.1186/s12890-022-02260-z)
Supplement: Supplementary file 2 — Additional file 2. SBT indications and obtaining the diaphragm ultrasound video and measurement protocol. [file 12890_2022_2260_MOESM2_ESM.docx]

**Supplementary file 1**

**Spontaneous breathing trial indications:**

All patients were enrolled in the study at the first spontaneous breathing trial (SBT). Patients who were intubated and mechanically ventilated for more than 24 hours were considered eligible for SBT if all of the following criteria were met: (a) clinical improvement in underlying acute respiratory failure; (b) adequate cough reflex; (c) no excess and/or purulent tracheobronchial secretions; (d) stable cardiovascular status (i.e. heart rate <120 beats/min; systolic blood pressure: 90-160 mmHg; no or minimal use of vasopressors, i.e. dopamine or more dobutamine <5μg/kg/min or norepinephrine <0.05 μg/kg/min); (e) stable metabolic status (ie, electrolytes and blood glucose within normal ranges, body temperature <38°C, hemoglobin ≥ 8-10 g/dL); (f) Sufficient oxygenation (ie fraction of inspiratory oxygen (FiO_2_) ≤ 0.5 or partial pressure of arterial oxygen/fraction of inspiratory oxygen (PaO_2_/FiO_2_) ≥ 150 mmHg, positive end expiratory pressure (PEEP) ≤8cmH_2_O, arterial oxygen saturation (SaO_2_) > 92%; (g) good lung function (ie, RR ≤ 30 beats/min, VT ≥ 5ml/kg ideal body weight (IBW), no significant respiratory acid intoxication; and (h) Richmond Anxiety and Sedation Scale scores between -1 and +1.

**Obtaining the diaphragm ultrasound video protocol**

The line array probe is placed between the right anterior axillary line and the midaxillary line, perpendicular to the eighth and ninth ribs where the zone of apposition of the diaphragm could be visualized. If the lungs obstruct the diaphragm during inspiration, the transducer can be moved forward along the intercostal space to the anterior axillary line, or along the anterior axillary line toward the foot, for the best image quality. Only one focus level was chosen, and the depth was optimized to see the diaphragmatic as clearly as possible during the entire inspiratory phase at maximum frame rate. The diaphragmatic ultrasound image quality can be enhanced by "rocking" and "sliding" to change the transducer position and angle. Finally, more than 6s of video of diaphragm movement was collected under these optimum conditions for off-line speckle-tracking analysis using EchoPacs (GE Healthcare, Milwaukee, MI).

**Conventional Diaphragmatic ultrasound indices measurement protocol**

M-mode ultrasound was used to assess the diaphragmatic excursion (DE), diaphragmatic thickness at end-inspiration (DTei), end-expiration (DTee), and diaphragmatic thickening fraction (DTF). The line array probe was placed between the right anterior axillary line and the midaxillary line, perpendicular to the eighth and ninth ribs, where the zone of apposition of the diaphragmatic could be observed. For m-mode imaging, the cursor line was equidistant between the two ribs and perpendicular to the diaphragm. Diaphragmatic thickness, excluding the pleura and outer peritoneum, was measured from the inner edge to the inner edge at the end of expiration and inspiration. DTF is calculated as: DTei minus DTee divided by DTee, expressed as a percentage: DTF = [(DTei – Dtee) / DTee] x 100%^1,2^. When measuring DE, a phased array probe was placed at the intersection of the right midclavicular and subcostal lines, with the transducer facing the skull and dorsal side. DE was calculated using m-mode imaging with the cursor line perpendicular to the diaphragmatic dome's most distal position, and it was estimated as the highest height change of the echo line on the m-mode image ^1,2^.

**Reference**

1. Orde SR, Boon AJ, Firth DG, Villarraga HR, Sekiguchi H. Diaphragm assessment by two dimensional speckle tracking imaging in normal subjects. *BMC Anesthesiol.* 2016;16(1):43.

2. Theerawit P, Eksombatchai D, Sutherasan Y, Suwatanapongched T, Kiatboonsri C, Kiatboonsri S. Diaphragmatic parameters by ultrasonography for predicting weaning outcomes. *BMC Pulm Med.* 2018;18(1):175.

**Title and legends for the supplementary video**

**Video 1** Video clip of the positioning of the region of interest for speckle tracking analysis of diaphragm contraction
